# Conjugated Polymeric Nanostructures as Potential Tools in DNA Nanotechnology: Interactions with the Double Helix

**DOI:** 10.1021/acsomega.5c11640

**Published:** 2026-04-27

**Authors:** Rayane M. de Oliveira, Caio H. V. da Silva, Mariana P. Brandão, Andreza G. S. Subtil, Márcio S. Rocha

**Affiliations:** Departamento de Física, 28120 Universidade Federal de Viçosa, Viçosa, Minas Gerais 36570-900, Brazil

## Abstract

We synthesized two types of conjugated polymeric nanoparticles (CPNs), made with MEH-PPV (poly­[2-methoxy-5-(2-ethylhexyloxy)-1,4-phenylenevinylene]) and PFD (poly­(9,9-di-n-dodecyl fluorenyl-2,7-diyl)), that significantly interact with double-stranded DNA. The binding between these nanostructures and the biopolymer was characterized via single-molecule force spectroscopy assays performed by optical tweezers and by direct visualization via atomic force microscopy imaging. The results show that both types of CPNs bind cooperatively outside the double helix, inducing bending, and with a considerably high equilibrium association constant, on the order of 10^6^ M^–1^. We thus demonstrate that conjugated polymeric nanostructures can strongly and cooperatively interact with double-stranded DNA using single-molecule force spectroscopy. These interactions may represent promising long-term perspectives for applications in DNA nanotechnology and functional nanomaterials, such as the design of DNA-based nanodevices and the development of new drug carriers based on functionalized nanoparticles for cancer chemotherapies, to cite a few.

## Graphical abstract

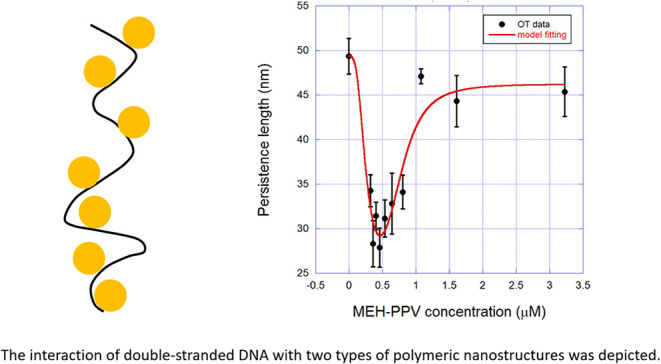

## Introduction

Complexes formed between nucleic acids and nanostructures of diverse types can present a large variety of functions and applications, such as in medical diagnostics and imaging,^1^ in the design of new biosensors and devices^1–3^ and in the construction of new carriers for drug delivery,^4–6^ to cite a few. The development of nanobiotechnology has intensified the investigation of interactions between functional nanomaterials and biomolecules. In particular, nanoparticles such as quantum dots (qdots) and conjugated polymeric nanostructures (CPNs) have allowed the development of many novel applications in the past years due to their unique optical, electronic, and physicochemical properties.^7–9^ Despite these advances, quantitative physicochemical characterization of the interactions between conjugated polymer nanostructures and nucleic acids at the molecular level remains relatively scarce, especially under near-physiological conditions and with single-molecule resolution. Such quantitative information is essential to better understand binding mechanisms, cooperativity effects, and the potential of these nanostructures for integration into DNA-based nanodevices and functional supramolecular assemblies.^10,11^

In this work, we study the formation of supramolecular structures between two different types of CPNs and single molecules of double-stranded DNA, which have been shown to be high-affinity substrates for the binding of our nanoparticles, synthesized here using the conjugated polymers MEH-PPV (poly­[2-methoxy-5-(2-ethylhexyloxy)-1,4-phenylenevinylene]) and PFD (poly­(9,9-di-n-dodecyl fluorenyl-2,7-diyl)).^12^ Rather than focusing solely on the existence of interactions between CPNs and DNA, this study aims to provide a quantitative single-molecule mechanical characterization of their binding affinity, cooperativity, and effects on the structural mechanics of the double helix.

Using single-molecule force spectroscopy performed with optical tweezers on the DNA–CPN complexes formed, we were able to characterize the changes in the mechanical properties of the double helix upon CPN binding. We show that, despite not altering the natural interspace between consecutive base pairs along the double helix, the nanoparticles induce bending upon binding, significantly modifying the effective persistence length of the complexes relative to that of the bare DNA molecule. These findings suggest that the CPNs bind outside the double helix in a similar way to what occurs with DNA groove binders, although in the present case, there is no fit to the grooves due to the much larger size of the CPNs.

Furthermore, the changes measured in the effective persistence length of the complexes formed as a function of the CPN concentration in the sample were used to deduce the physical chemistry of the effective binding, allowing the determination of important parameters such as the equilibrium binding association constant and the cooperativity degree of the interaction. Such quantitative characterization contributes to a broader understanding of nano-bio interfaces involving conjugated polymer nanostructures and nucleic acids, an area of growing interest for the design of functional nanomaterials and DNA-based nanotechnological systems.^10,11,13,14^

The interactions between DNA and the two types of CPNs used were also confirmed here by a second independent technique: direct imaging of the complexes formed in atomic force microscopy assays. These experiments allowed the visualization of the bound nanoparticles along the double helix, confirming the binding.

Overall, this work provides a detailed physicochemical and single-molecule perspective on the interaction between conjugated polymeric nanostructures and DNA. These results open the door for the use of CPNs to construct supramolecular assemblies with nucleic acids for various purposes, helping to bridge the gap between the growing use of these materials in nanobiotechnology and the need for a quantitative understanding of their molecular-level interactions with nucleic acids. Such interactions can be important in the future design of DNA-based nanodevices and in the development of new drug carriers based on functionalized nanoparticles for cancer chemotherapies, to cite a few.^15–20^

## Materials and Methods

### CPNs Synthesis and Characterization

Conjugated polymer nanoparticles (CPNs) were synthesized using the reprecipitation^7^ method from the conjugated polymers MEH-PPV (poly­[2-methoxy-5-(2-ethylhexyloxy)-1,4-phenylenevinylene]) and PFD (poly­(9,9-di-n-dodecylfluorenyl-2,7-diyl)). The polymers were first dissolved in tetrahydrofuran (THF) at a concentration of 1.0 g/L. Then, 1.0 mL of the polymer solution was rapidly injected into 10 mL of deionized water under constant magnetic stirring for 5 min. The resulting dispersion was subsequently subjected to ultrasonic treatment for 10 min. Finally, the nanoparticle suspension was vacuum-filtered by using a paper filter with a pore size of 2.5 μm to isolate the MEH-PPV and PFD nanoparticles.

Optical characterizations (UV–visible absorption spectra and fluorescence spectra) were obtained using the HORIBA Fluoromax-plusC spectrometer, placing the solution in quartz cuvettes at room temperature for all samples.

The morphology of the CPNs was characterized using a Field Emission Gun Scanning Electron Microscope (FEG-SEM, Tescan Mira). Samples of both nanoparticle solutions were prepared using 20 μL, which were deposited on silicon substrates and allowed to dry completely. Afterward, a thin layer of copper was sputter-deposited on the samples, enabling their visualization with the FEG-SEM.

The hydrodynamic mean diameter of the nanoparticles was also obtained through dynamic light scattering (DLS) using a Zetasizer Nano S device (Malvern Instruments). The measurements were conducted in polystyrene cuvettes at a controlled temperature of (25.0 ± 0.1) °C. We measured the scattered intensity at a detection angle of 173° relative to the incident 4 mW He/Ne laser beam with a wavelength of 632.8 nm. The CONTIN algorithm, integrated within the instrument’s software, was employed to analyze the intensity autocorrelation functions and ascertain the size distribution. Each reported value is the average of five sets of ten measurements accumulated over a 90-s period. This process was repeated five times to ensure accuracy, and we obtained its average and standard deviation.

### Sample Preparation and Experimental Procedure for Optical Tweezers Assays

The samples prepared for the single-molecule force spectroscopy assays consist of biotin-labeled λ-DNA (48,502 base pairs, New England Biolabs) attached at the ends to a streptavidin-coated polystyrene bead with a 3 μm diameter (Bangs Laboratories) and to a streptavidin-coated glass coverslip (which serves as the bottom surface of the sample chamber used).^21^ The samples are prepared with bare DNA molecules and beads in a Phosphate Buffered Saline (PBS) buffer, [Na^+^] = 150 mM, without nanoparticles. Before adding any CPNs to the sample, a bare DNA molecule is chosen and stretched with the tweezers 5 to 10 times to test its integrity. To do this, the force–extension curves of each run are collected and fitted to the Marko-Siggia Worm-Like Chain (WLC) model^22^ to determine the two basic mechanical parameters: the contour length and the persistence length. If the average values obtained for these parameters agree with those expected for a bare λ-DNA molecule under nearly physiological buffer conditions (contour length *L* ∼ 16.5 μm, persistence length *A* ∼ 50 nm), then the experiment proceeds using this molecule, introducing the nanoparticles into the sample.

The CPNs are introduced at the desired concentration in the sample chamber, and the force spectroscopy assays are repeated for various nanoparticle concentrations, gradually increasing these concentrations and collecting the values of the mechanical parameters at each fixed CPN concentration, always using the same DNA molecule to guarantee accurate results. For each CPN concentration, at least 5 independent measurements were performed with the same DNA molecule. Indeed, the average results reported in the following section for the mechanical parameters as a function of the CPN concentration in the sample were obtained by performing repeated stretching assays under the same conditions with the same DNA molecule (5 to 10 independent measurements), and the error bars were calculated as the mean standard error from these independent measurements. For each nanoparticle concentration used, we waited 30 min for the DNA–CPN to reach equilibrium before performing the measurements. Such a procedure allowed us to obtain reliable results of the mechanical parameters (contour and persistence lengths) as a function of the CPN concentration. The entire experiment was also repeated using different samples (5 different DNAs for each type of CPN) to check reproducibility. Although the number of independent DNA molecules used appears small in principle, it is worth emphasizing that our results are very consistent, always within the error bars, even when changing the molecule used.

Details on the sample preparation and measurement procedures can be found in previous references.^21,23^

### Determining the Physicochemical (Binding) Parameters of the Binding Modes

The persistence length *A* of a DNA–ligand complex as a function of the ligand concentration in the sample can be modeled using a quenched-disorder statistical model for ligand binding, previously developed in our group.^24^ For the simpler cases (monotonic increase or decrease of the persistence length), the model can be stated as^21,25^

1 $$\frac{1}{A}=\frac{1-r/r_{\max}}{A_{0}}+\frac{r/r_{\max}}{A_{1}}$$

where *r* is the bound ligand fraction, *r*_max_ is its saturation value, *A*_0_ is the bare DNA persistence length, and *A*_1_ is the persistence length at bound ligand saturation.

The bound ligand fraction *r* can be connected to an appropriate binding isotherm that describes the binding. For noncooperative binding processes, the McGhee–von Hippel classic model^26^ can be used.^21,25^ For cooperative binding processes, on the other hand, the Hill isotherm has been proven to be a good choice to describe the physical chemistry of the interaction.^21,25^ Such isotherm reads

2 $$\frac{r}{r_{\max}}=\frac{{[K(C_{T}-rC_{bp})]}^{n}}{1+{[K(C_{T}-rC_{bp})]}^{n}}$$

where *K* is the association equilibrium binding constant, *C*_*T*_ is the ligand total concentration in the sample, *C*_*bp*_ is the DNA base-pair concentration in the sample, and *n* is the Hill exponent, a parameter that accounts for the degree of cooperativity in the binding reaction. If *n* > 1, the binding is positively cooperative, and a bound ligand facilitates subsequent ligand binding. If *n* < 1, the binding is negatively cooperative, and a bound ligand hinders subsequent ligand binding. Finally, if *n* = 1, the binding is noncooperative, and the ligands bind independently along the double helix.

For more complex DNA ligands that induce nonmonotonic behaviors on the persistence length, the model stated in eq 1 can be reformulated considering first-neighbor interactions, reading

3 $$\frac{1}{A}=\frac{1}{A_{0}}+\left(\frac{2}{A_{1}}-\frac{2}{A_{0}}\right)\frac{r}{r_{\max}}+\left(\frac{1}{A_{0}}-\frac{2}{A_{1}}+\frac{1}{A_{2}}\right){\left(\frac{r}{r_{\max}}\right)}^{2}$$

and *r* can be expressed by a convenient binding isotherm as well.

In any case, fitting the experimental data of the persistence length as a function of the ligand concentration in the sample, by using this type of model, allows one to determine the binding parameters that characterize the interaction.

### Atomic Force Microscopy of DNA–CPN Complexes

Atomic force microscopy imaging of the DNA complexes formed with the two types of CPNs was also performed in order to confirm the binding of such nanoparticles to the double helix. The setup used in these experiments was an NT-MDT NTEGRA PRIMA operating in tapping mode with TAP300 Al-G tips (Budget Sensors).

The samples were prepared for these assays using 3 kbp DNA molecules (ThermoFisher Sci.) in a 10 mM Tris-HCl buffer with the addition of 100 mM MgCl_2_ (pH 8.0), which is needed to attach DNA molecules to the mica substrates. The CPNs were then added to these solutions and allowed to equilibrate with DNA for ∼30 min. After that time, 20 μL of the desired solution was deposited on the substrate and completely dried out with nitrogen at ambient temperature (∼25 °C).

All experiments were performed in air, at ambient temperature, and with humidity around 50%. We obtained the average diameter and its standard deviation for each image, selecting at least 10 bare DNA and DNA complexes using the software Gwyddion. This experimental procedure has been proven reliable for visualizing deposited bare DNA molecules and the complexes formed between the biopolymer and ligands.^27–31^

## Results and Discussion

### CPN Characterization

Figure 1 shows the typical normalized absorption and fluorescence spectra of the conjugated polymer nanoparticles obtained for both polymers. In panel (a), the results for MEH-PPV CPNs are presented, while in panel (b), the results for PFD CPNs are shown. The differences in these optical spectra are very significant, as expected, due to the distinct optical properties of the two types of polymers used to synthesize the nanoparticles.

**Figure 1:**
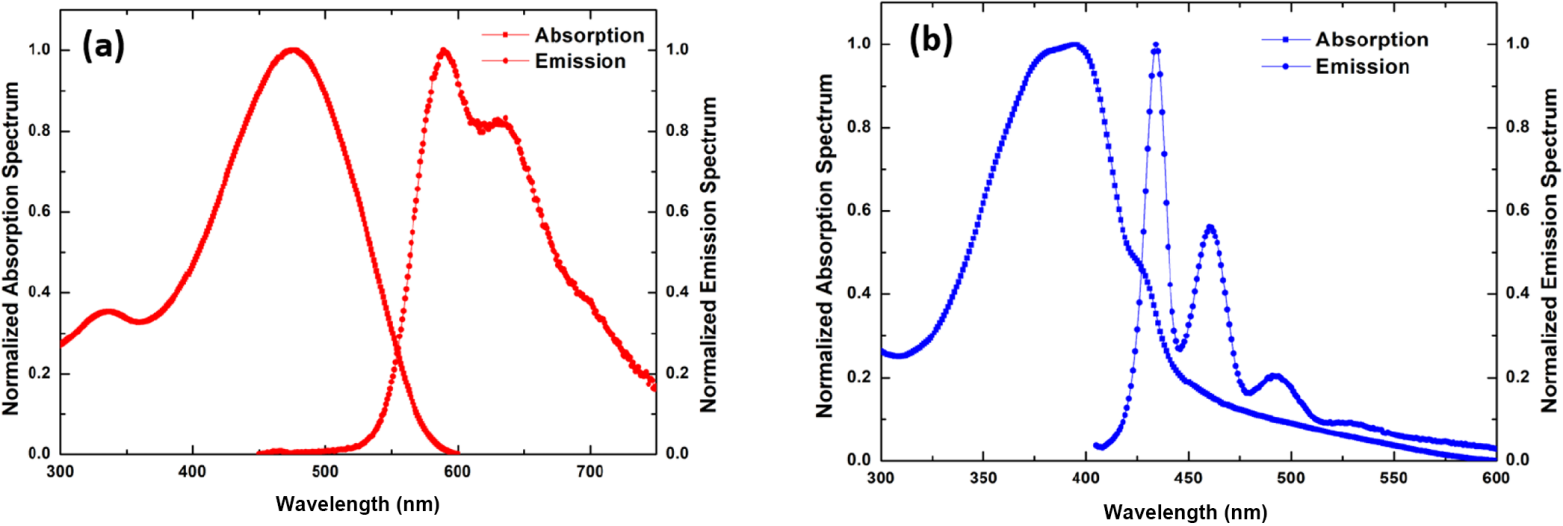
Typical normalized absorption and fluorescence spectra: (a) MEH-PPV CPNs and (b) PFD CPNs. Excitation: 401 nm.

Figure 2 shows typical FEG-SEM images of the two types of CPNs deposited on silicon substrates using the procedure described in the previous section. From such images, it is clear that the two types of CPNs present very different average sizes on dry substrates: while for the MEH-PPV CPNs (panel (a)) we obtain an average diameter on the order of ∼100 nm, the PFD CPNs are typically three or more times smaller.

**Figure 2:**
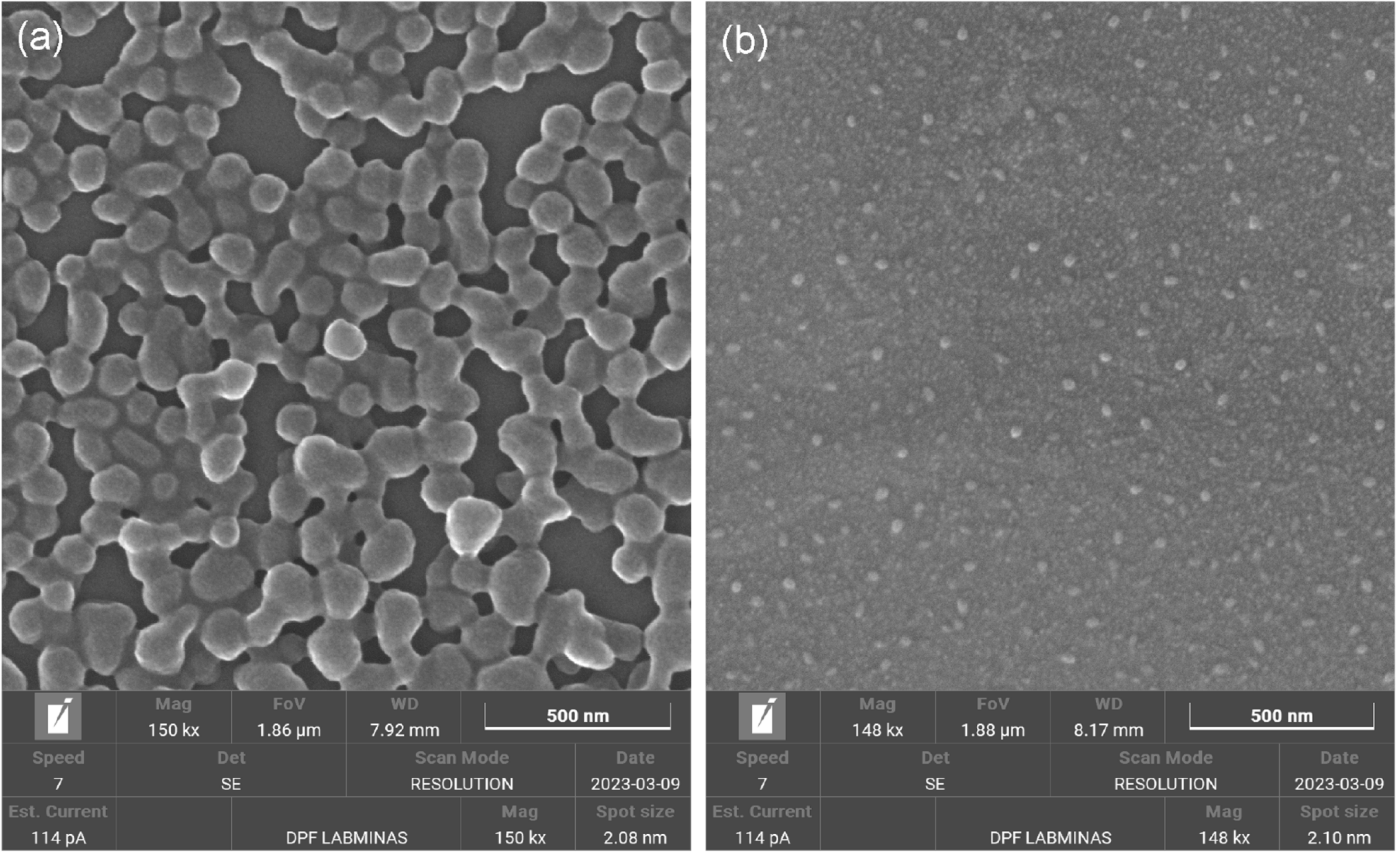
Typical Field Emission Gun Scanning Electron Microscopy (FEG-SEM) images of the two types of CPNs deposited on silicon substrates: (a) MEH-PPV CPNs and (b) PFD CPNs.

Interestingly, Dynamic Light Scattering (DLS) measurements revealed that the average hydrodynamic diameters of both types of CNPs in water were similar, ranging from 100 to 150 nm depending on the polymer concentration.^12^ The discrepancy between the average diameter from FEG-SEM results and the average hydrodynamic diameter from DLS results was explained earlier on the basis of the hydration shell around the nanoparticles in each case.^12^

### Optical Tweezers Assays

In Figure 3, we show the average contour (panels (a) and (b)) and persistence (panels (c) and (d)) lengths obtained for the two types of complexes studied, DNA–MEH-PPV and DNA–PFD, as a function of the CPN concentration in the sample.

**Figure 3:**
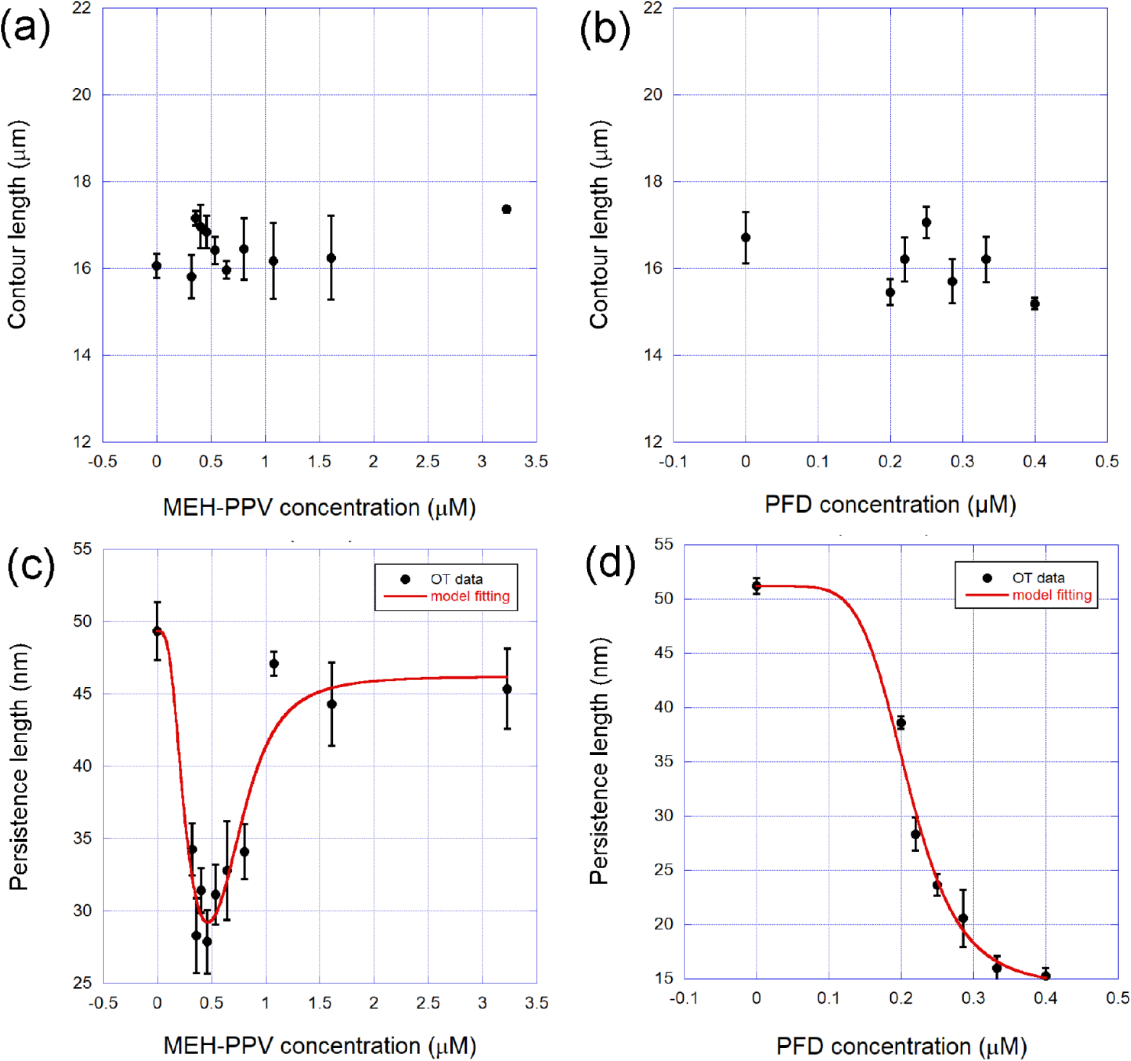
Mechanical parameters of the complexes formed between DNA and the two types of CPNs in solution as a function of the CPN concentration in the sample: (a) Contour length of the DNA–MEH-PPV complexes. (b) Contour length of the DNA–PFD complexes. (c) Persistence length of the DNA–MEH-PPV complexes. (d) Persistence length of the DNA–PFD complexes. The red lines shown in panels (c) and (d) are fittings to the quenched-disorder model of the persistence length, from which the binding parameters can be determined.

Observe that none of the CPNs change the DNA contour length upon binding, which means that the interactions do not change the average interspace between consecutive base pairs in the double helix. The persistence length of the complexes formed changes drastically with the nanoparticle concentration, confirming that there is indeed an effective interaction between the two types of CPNs and the DNA double helix.

In the case of the MEH-PPV CPN, panel (c), the persistence length exhibits a more complicated nonmonotonic behavior, first decreasing for low concentrations (<0.5 μM) and then increasing until saturation. For the PFD CPNs, panel (d), the behavior is simpler: a monotonic decrease as a function of the CPN concentration from the bare DNA value until saturation. Such a difference can be explained based on the difference in size between the two types of CPNs: both tend to induce bendings on the double helix, first decreasing the persistence length. However, due to their considerably greater size, MEH-PPV CPNs are expected to induce volume-exclusion effects at higher concentrations, increasing the persistence length—which is exactly what is shown in Figure 3, panel (c).

The red lines shown in panels (c) and (d) of Figure 3 fit the quenched-disorder model presented in the former section, which predicts the behavior of the effective persistence length of the complexes formed as a function of the ligand concentration in the sample.

In the case of MEH-PPV, the two-site version of the quenched-disorder model was used, as the behavior presented by the persistence length is nonmonotonic. The Hill binding isotherm was employed in the model to determine the effective physicochemical parameters of the interaction, allowing one to obtain information about the binding affinity and cooperativity. In the case of PFD CPNs, the simpler one-site version of the model was utilized, as the persistence length decays monotonically, and the Hill binding isotherm was also applied to compare the binding parameters of the two ligands with accuracy.

In Table 1 we show the results obtained from the persistence length fittings for the two CPNs. Observe that both exhibit an equilibrium binding association constant (*K*) with DNA on the order of 10^6^ M^–1^, indicating a considerably strong binding, compatible with the affinity of many DNA groove binders (typically ∼10^6^ to 10^8^ M^–1^) and intercalators (typically ∼10^4^ to 10^6^ M^–1^).^21,32^ Furthermore, both nanoparticles exhibited a Hill exponent considerably greater than unity, indicating positive cooperativity upon binding.^21,32^ For comparison purposes, cooperative groove binders usually present a Hill exponent between 2 and 4, while intercalators, in general, do not cooperate when binding to DNA (Hill exponent ∼1).^21,32^ Finally, the values obtained for the local persistence lengths, *A*_1_ and *A*_2_ reflect the local changes induced in the bending rigidity of the double-helix upon CPN binding.

**Table 1:** Binding Parameters Obtained from the Persistence Length Fittings Performed with the Optical Tweezers Data for the MEH-PPV and PFD CPNs Interaction with Double-Stranded *λ*-DNA

	MEH-PPV	PFD
*K* (× 10^6^ M^–1^)	2.8 ± 0.3	3.8 ± 0.5
*n*	3.6 ± 0.3	5.9 ± 0.7
*A*_1_ (nm)	21 ± 2	14.2 ± 0.3
*A*_2_ (nm)	46 ± 5	-

### Atomic Force Microscopy (AFM) Assays

In Figure 4 we show typical atomic force microscopy (AFM) images obtained for the DNA complexes formed with both types of CPNs, deposited on mica substrates and scanned as discussed in the previous section. These images confirm that both nanoparticles (brighter dots in the images) bind to the double helix. Furthermore, we measured the average diameters of the double helix and the bound nanoparticles to confirm the nature of these samples. Since AFM imaging was performed on dried samples in air, structural rearrangements and aggregation effects may occur. Therefore, AFM results should be interpreted as a qualitative confirmation of binding rather than quantitative structural evidence. In contrast, the optical tweezers measurements provide solution-based quantitative characterization of the interaction. The results are shown in Table 2.

**Figure 4:**
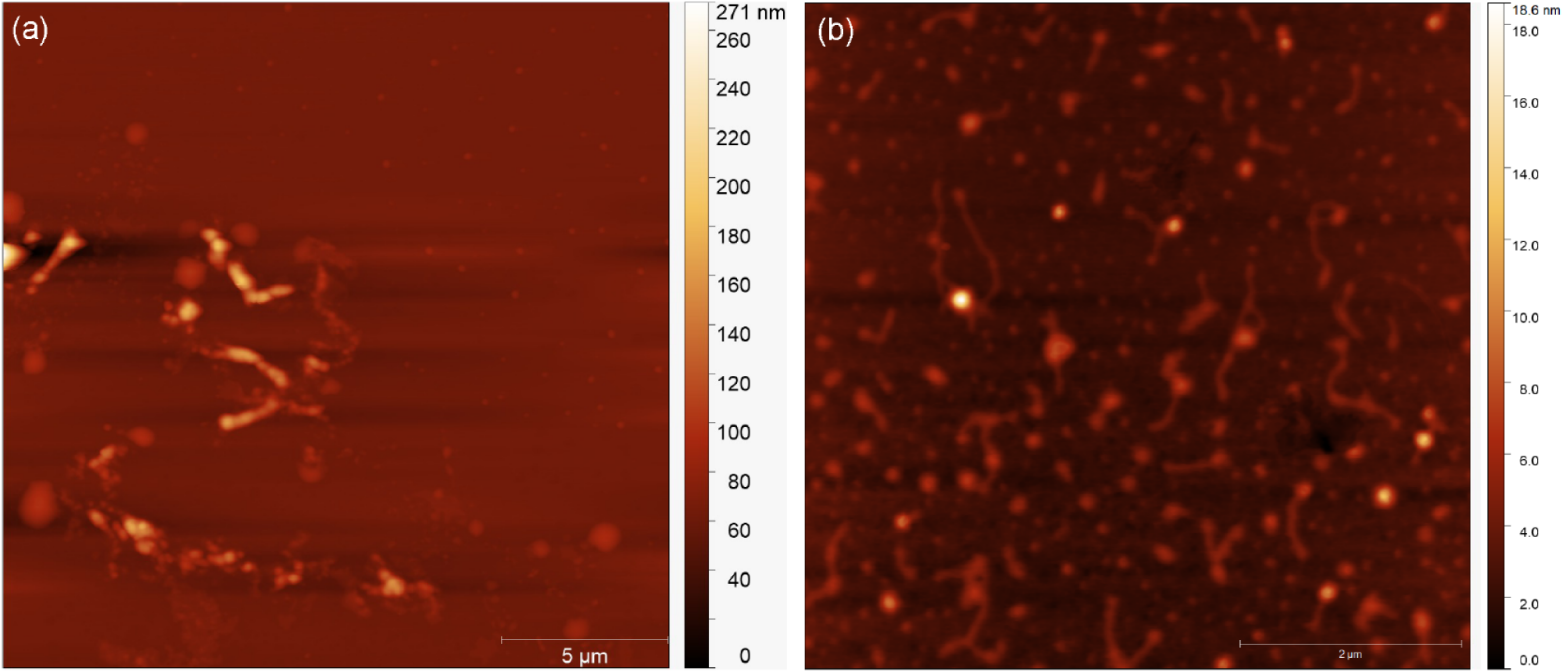
Typical AFM images of the DNA complexes formed with the two types of CPNs, deposited on mica substrates: (a) MEH-PPV CPNs and (b) PFD CPNs.

**Table 2:** Average Diameter and Standard Deviation of DNA and Bound MEH-PPV and PFD CPNs Obtained from AFM Images

	DNA	MEH-PPV	PFD
Diameter (nm)	2.7 ± 0.2	106 ± 2	6.9 ± 0.6

Observe that the measured diameter of the double helix is within the expected range, around ∼2 nm. The diameter measured for the bound MEH-PPV and PFD CPNs also agrees with the values obtained from the FEG-SEM images of these isolated nanoparticles (i.e., without DNA), confirming that the visualized bright dots bound to the double helix are indeed these CPNs. Thus, our AFM assays complement and confirm the results obtained from the optical tweezers experiments, showing that our CPNs are really capable of binding to DNA.

In summary, here we report, for the first time, a single-molecule mechanical quantification of binding affinity and cooperativity of conjugated polymeric nanostructures to the DNA double helix, opening the door for the use of such structures in DNA nanotechnology. In particular, since DNA is an important target for anticancer drugs inside cells and nanoparticles are widely employed today in the design of drug carriers, our CPNs appear to show strong potential for the construction of drug carriers.

## Conclusions

We synthesized and characterized two types of conjugated polymeric nanoparticles (MEH-PPV and PFD) that exhibited a strong potential to interact with the DNA double helix under physiological conditions. The mechanical changes in the double helix and the physical chemistry of the interactions were determined, showing that the CPNs bind cooperatively outside the double helix in a similar way to what occurs with DNA groove binders, although in the present case, there is no fit to the grooves due to the much larger size of the nanoparticles—a result that should be confirmed in the future using complementary techniques. The effective interactions induce bending in the biopolymer and exhibit strong equilibrium binding constants on the order of 10^6^ M^–1^. The binding of both CPNs was also confirmed by directly imaging the DNA-CPN complexes formed in atomic force microscopy assays. These results demonstrate that conjugated polymeric nanostructures can strongly and cooperatively interact with double-stranded DNA, as quantitatively revealed by single-molecule force spectroscopy. While the present study is focused on fundamental physicochemical characterization, such interactions may represent promising long-term perspectives for applications in DNA nanotechnology and functional nanomaterials.

## References

[1] Wang T.-H. (2013). Discerning
single molecule interactions of DNA and quantum dots. Biotechnol. J..

[2] Hua Y., Ma J., Li D., Wang R. (2022). DNA-Based Biosensors for the Biochemical
Analysis: A Review. Biosensors.

[3] Wang Q., Gao F., Ni J., Liao X., Zhang X., Lin Z. (2016). Facile construction
of a highly sensitive DNA biosensor by in-situ assembly of electro-active
tags on hairpin-structured probe fragment. Sci.
Rep..

[4] Panda P., Mohapatra R. (2025). Advancements
in DNA nanotechnology for targeted drug
delivery: Design strategies and applications. Hybrid Adv..

[5] Kumar M., Jha A., Mishra B. (2024). DNA-Based
Nanostructured Platforms as Drug Delivery
Systems. Chem. Bio Eng..

[6] Guan C., Zhu X., Feng C. (2021). DNA Nanodevice-Based
Drug Delivery Systems. Biomolecules.

[7] Pecher J., Mecking S. (2010). Nanoparticles of conjugated
polymers. Chem. Rev..

[8] Wu C., Chiu D. T. (2013). Highly fluorescent
semiconducting polymer dots for
biology and medicine. Angew. Chem..

[9] Kang S., Yoon T. W., Kim G.-Y., Kang B. (2022). Review of Conjugated
Polymer Nanoparticles: From Formulation to Applications. ACS Appl. Nano Mater..

[10] Cagnetta G. E., Martínez S. R., Ibarra L. E., Wendel A., Palacios R. E., Chesta C. A., Gómez M. L. (2025). Photoactive broad-spectrum dressings
with antimicrobial and antitumoral properties. Biomat. Adv..

[11] Pradhan T., Chelike D. K., Roy D., Pramanik T., Dolui S. (2025). Stimuli-Responsive
Multiacceptor Conjugated Polymers: Recent Trend and Future Direction. ACS Polym. Au.

[12] de
Lana Junior M. L., da Silva C. H. V., Brandão M. P., da Silva Subtil A. G. (2025). Conjugated polymer nanoparticles of Poly­(9,9-di-n-dodecylfluorene-2,7-diyl)
and MEH-PPV: Photoluminescence properties, *β*-phase formation and morphology. J. Fluor..

[13] Caverzan M. D., Vasconsuelo A. B. M., Cerchia L., Palacios R. E., Chesta C. A., Ibarra L. E. (2025). Preclinical
Toxicological Characterization of Porphyrin-Doped
Conjugated Polymer Nanoparticles for Photodynamic Therapy. Pharmaceutics.

[14] Modicano P., Trutschel M.-L., Phan-Xuan T., Matarése B. F.
E., Urbano L., Green M., Mäder K., Dailey L. A. (2025). Does Encapsulation
of *π*-Conjugated
Polymer Nanoparticles within Biodegradable PEG–PLGA Matrices
Mitigate Photoinduced Free Radical Production and Phototoxicity?. Adv. Therap..

[15] Patra J. K., Das G., Fraceto L. F., Campos E. V. R., Rodriguez-Torres M. D.
P., Acosta-Torres L. S., Diaz-Torres L. A., Grillo R., Swamy M. K., Sharma S. (2018). Nano based
drug delivery systems: Recent developments and future prospects. J. Nanobiotechnol..

[16] Kaul G., Amiji M. (2005). Tumor-targeted gene
delivery using poly­(ethylene glycol)-modified
gelatin nanoparticles: in vitro and in vivo studies. Pharm. Res..

[17] Schiffelers R. M., Ansari A., Xu J., Zhou Q., Tang Q., Storm G. (2004). Cancer siRNA therapy
by tumor selective delivery with
ligand-targeted sterically stabilized nanoparticle. Nucleic Acids Res..

[18] Brannon-Peppas L., Blanchette J. O. (2004). Nanoparticle and targeted systems for cancer therapy. Adv. Drug Delivery Rev..

[19] Kaul G., Amiji M. (2004). Biodistribution and targeting potential
of poly­(ethylene glycol)-modified
gelatin nanoparticles in subcutaneous murine tumor model. J. Drug Target.

[20] Farokhzad O. C., Cheng J., Teply B. A., Sherifi I., Jon S., Kantoff P. W. (2006). Targeted nanoparticle-aptamer bioconjugates
for cancer chemotherapy in vivo. Proc. Natl.
Acad. Sci. U. S. A..

[21] Rocha, M. S. DNA Interactions with Drugs and Other Small Ligands - Single Molecule Approaches and Techniques; 1st ed.; Academic Press (Elsevier), 2023.

[22] Marko J. F., Siggia E. D. (1995). Stretching DNA. Macromolecules.

[23] Oliveira L., Rocha M. S. (2017). Force spectroscopy unravels the role of ionic strength
on DNA-cisplatin interaction: Modulating the binding parameters. Phys. Rev. E.

[24] Siman L., Carrasco I. S. S., da Silva J. K. L., Oliveira M. C., Rocha M. S., Mesquita O. N. (2012). Quantitative Assessment of the Interplay between DNA-Elasticity
and Cooperative Binding of Ligands. Phys. Rev.
Lett..

[25] Rocha M. S. (2015). Extracting
physical chemistry from mechanics: A new approach to investigate DNA
interactions with drugs and proteins in single molecule experiments. Integr. Biol..

[26] McGhee J. D., von Hippel P. H. (1974). Theoretical aspects of DNA-protein interactions - cooperative
and non-cooperative binding of large ligands to a one-dimensional
homogeneous lattice. J. Mol. Biol..

[27] Rivetti C., Guthold M., Bustamante C. (1996). Scanning Force
Microscopy of DNA
Deposited onto Mica: Equilibration versus Kinetic Trapping Studied
by Statistical Polymer Chain Analysis. J. Mol.
Biol..

[28] Cesconetto E. C., Junior F. S. A., Crisafuli F. A. P., Mesquita O. N., Ramos E. B., Rocha M. S. (2013). DNA Interaction with Actinomycin D: Mechanical Measurements
Reveal the Details of the Binding Data. Phys.
Chem. Chem. Phys..

[29] Lin Z., Wang C., Feng X., Liu M., Li J., Bai C. (1998). The observation of the local ordering characteristics of spermidine-condensed
DNA: Atomic force microscopy and polarizing microscopy studies. Nucleic Acids Res..

[30] Hansma H. G., Golan R., Hsieh W., Lollo C. P., Mullen-Ley P., Kwoh D. (1998). DNA condensation for gene therapy as monitored by atomic force microscopy. Nucleic Acids Res..

[31] Saito M., Kobayashi M., Iwabuchi S., Morita Y., Takamura Y., Tamiya E. (2004). DNA Condensation Monitoring After Interaction with
Hoechst 33258 by Atomic Force Microscopy and Fluorescence Spectroscopy. J. Biochem..

[32] Bazoni R. F., Moura T. A., Rocha M. S. (2020). Hydroxychloroquine Exhibits a Strong
Complex Interaction with DNA: Unraveling the Mechanism of Action. J. Phys. Chem. Lett..

